# Rural doctor quota students in Germany – who are they? Data on first year students from two cohorts in the federal state of Saxony

**DOI:** 10.1080/10872981.2025.2497325

**Published:** 2025-05-06

**Authors:** Anne-Kathrin Geier, Anja Heuser, Markus Bleckwenn, Tobias Deutsch

**Affiliations:** Institute of General Practice, Faculty of Medicine, University of Leipzig, Leipzig, Germany

**Keywords:** Undergraduate medical education, general practice, rural health services, medical students, public health

## Abstract

The lack of physicians in rural areas is a universal problem. To increase the attractiveness of rural practice for medical students, the contribution of medical schools is undisputed. However, much of the evidence on interventions before and during undergraduate education comes from countries with large areas and low population density like Australia and Canada. In Germany, selective admission to medical studies for students who agree to become rural general practitioners is still a new concept. The aim of this study was to assess the sociodemographic characteristics, attitudes and career aspirations of the rural doctor quota students from one medical school in Germany compared to their non-quota counterparts. For this cross-sectional study, a paper-based anonymous questionnaire was distributed to all first year medical students at Leipzig University in two consecutive study years.Descriptive analyses and group differences were calculated using SPSS. The response rate was 87.3% with *n* = 604 completed questionnaires and 40 (6.6%) students self-classified as rural doctor quota students. Quota students grew up in rural areas significantly more often than their counterparts and had more working experience in the medical field. General practice was the preferred career option for 64.1% (25/39, versus 2.7% [15/549] of non-quota students). Working self-employed in one’s own medical practice was the preferred option for 71.1% (27/38) of quota students (vs. 28.0% [153/546] of non-quota students). Quota students valued a broad spectrum of patients, a long-term doctor–patient relationship, employee management and prestige more highly than their fellow students. Students from the rural doctor quota largely exhibit characteristics and attitudes that are compatible with future rural practice, despite showing little differences in sociodemographic items such as age and marital status. Not all students agree with the program objective. To demonstrate an impact on the health services, longitudinal data is necessary to monitor career choices over time.

## Background

Unequal access to healthcare due to a lack of physicians in rural areas is a universal problem [1–3]. For this reason, enormous efforts have been made in many parts of the world to secure primary care in rural areas. Approaches to the problem include political and financial incentives, increasing the overall number of physicians, and interventions during undergraduate and postgraduate medical training [4–6]. Medical schools can contribute to the recruitment of rural doctors in various ways. There is now broad evidence, particularly from Australia, Canada, and the United States, that rural clinical experiences, rurally focused curricula, and rural campuses can increase the number of graduates choosing a career in rural areas [1,2,5,7–9]. Furthermore, the medical students’ origin has a significant impact. There are various studies that show the increased likelihood of students with a rural background becoming a rural doctor compared with their fellow students from urban areas [1,10–13]. Consequently, the selection of students with specific characteristics is a starting point for increasing the number of graduates becoming rural doctors.

In Germany, medical education lasts 6 years. Detailed information about the study program has been published by Chenot [14]. Program slots for medical students are largely allocated centrally. Depending on the university, different weightings are given to high school diploma grades, aptitude tests, existing vocational training, and interviews [15]. The process is highly competitive with more than three times as many applicants as study places available [16]. Medical Schools are usually public and located in urban agglomerations with over 100,000 inhabitants. Undergraduate medical training mainly takes place at affiliated university hospitals. Rural primary care in Germany, on the other hand, mainly occurs in individual outpatient practices, with self-employment in one‘s own practice being the predominant model (both for GPs and specialists). Remuneration is primarily based on a quarterly flat rate with complex individual service remuneration [17]. Rural GP practices are increasingly unable to be filled, so that according to a current report 11,000 doctor’s posts could be vacant by 2035 [18]. At the same time, the shortage of (rural) doctors has long affected other specialties as well.

To counter the increasing shortage of GPs in rural areas, since 2019 10 out of 16 federal German states have introduced a rural doctor quota [19]. Students willing to work in rural areas later on can opt for a separate application procedure. This increases the chances of getting one of the places reserved for this purpose for applicants who meet certain requirements. At the same time, applicants commit to working as general practitioners (GPs) for 10 years in underserved rural areas of their federal state. Failure to fulfill the contract results in contractual penalties of up to 250 000 euros [20]. In most federal states, between 4% and 10% of study places are allocated according to the rural doctor quota [21]. In absolute figures, this currently corresponds to between 25 places for first-year students in Saxony-Anhalt [22] and 113 places in Bavaria [23], for example. As medical studies in Germany take 6 years, no data on graduates from Germany is available yet.

In the federal state of Saxony, 40 places per year have currently been allocated at three different study locations via the rural doctor quota since 2022 [24]. Of them, the University of Leipzig enrolls 22 students per year.

The selection procedure in Saxony is carried out by the state directorate [25]. The procedure has two stages. High school diploma grades and aptitude tests play a role in the first stage alongside work experience and voluntary services. The second step consists of standardized, structured interviews [26]. Here, the following items are assessed: ’1. subject-specific personal aptitude for working as a GP in the Free State of Saxony, 2. commitment to people, 3. social competence, 4. solution orientation and 5. analytical thinking’ [27]. Rural origin is not a criterion but might be included in the suitability assessment formed by the second step.

Apart from the admission procedure, the study program of the quota students does not differ from that of their fellow students.

Due to the novelty of the rural doctor quota in Germany and additional data protection issues, little is known about these students, their characteristics and attitudes at study entry in Saxony and other federal states [28]. As part of the Institute of General Practice’s survey of first-year students during the introductory week of medical studies at the University of Leipzig, first-year students have been asked about their characteristics and attitudes for almost a decade. This survey was expanded with the introduction of the rural doctor quota with the aim of finding out more about the quota students’ characteristics, whether they exhibit typical properties of future rural doctors and to what extent they differ from the rest of the group. Information on the characteristics and profiles of quote students is important to enable targeted study support and promotion, to optimize selection processes and to monitor the long-term success of the rural doctor quota program.

## Methods

### Sampling and design

A cross-sectional survey was carried out that included all first-year medical students at the University of Leipzig who started their medical studies in October 2022 (first cohort) and October 2023 (second cohort). During a formal introductory event students were verbally informed that participation in the survey was voluntary and anonymous and that their participation or non-participation was not associated with any advantages or disadvantages. Students who decided to take part completed the paper-based questionnaire and its return at the end of the introductory session was considered consent to take part.

### Questionnaire

The questionnaire was self-developed by a team of medical education researchers, based on extensive literature research and their own research experience. The questionnaire is part of a longitudinal research project running since 2016 and data from this instrument has been analysed for other projects [29].

The questionnaire consisted of three parts: sociodemographic information, future career plans and the weighting of different aspects in relation to a future medical career. In the second part, three career paths could be specifically rated as ’the favored career option’, ‘an imaginable career option’ or ‘no career option’: working as a GP, working in the outpatient sector, and working (self-employed) in their own practice. In a free-text question, students were also asked to indicate in a ranking list which specializations they are currently considering. Students could then choose if they could imagine working in a big city, a small town or a rural area (multiple answers possible). In the third part, 16 preferences regarding the future work as a medical specialist, which are either positively or negatively associated with GP career choice according to current scientific literature [30,31], could be rated on a five-point Likert-scale from ‘unimportant’ to “very important (e.g., ‘prestige’, ‘high income’, ‘intellectual challenge from daily work’, ‘broad spectrum of patients’). An English translation of the questionnaire used can be found in Appendix 1.

### Statistical analysis

Data were analysed using IBM SPSS Statistics 29.0. Frequencies were presented as %_valid_ (n_absolute_/n_valid_) considering missing values for single items. Continuous variables were presented as mean ± standard deviation (SD). Besides descriptive statistics group differences based on the variable ‘rural doctor quota student’ versus ‘non-quota student’ (independent variable) were examined using chi-square tests for frequencies and Mann-Whitney U-tests for central tendency due to the absence of normal distribution (Kolmogorov-Smirnov-Test) in the independent groups. We were interested in potential differences regarding sociodemographic information, future career plans and the weighting of different aspects in relation to a future medical career (dependent variables) without directed hypotheses due to the exploratory approach of our study. Statistical significance was assumed at *p* < 0.05.

## Results

In 2022 and 2023, a total of 692 students began their medical studies at the University of Leipzig (2022: 349 students, 2023: 343 students). The response rate was 87.3% with n = 604 returned and completed questionnaires, of which 40 (6.6%) students self-classified as students in the rural doctor quota. Since 22 quota students are enrolled each year, this represents 90.9% of 44 quota students in these two cohorts.

A socio-demographic description of all participants can be found in Table 1. Rural doctor quota students did not differ significantly from their fellow students in terms of their gender composition, their relationship status and in terms of having children. Similarly, differences in being a physician’s child and having a parent with a university degree were not significant.

**Table 1.** Sociodemographic data of quota and non-quota students.

	Non-quota students (*n* = 564)	Rural doctor quota students (*n* = 40)	Total (*n* = 604)	*P* value
	%n_valid_ (n/n_valid_)*	%n_valid_ (n/n_valid_)*	%n_valid_ (n/n_valid_)*	
Female gender	70.9 (392/553)	67.5 (27/40)	70.7 (419/593)	*p* = 0.178
Age (mean ± SD)	19.84 ± 2.08	20.85 ± 1.83	20.45 ± 2.30	***p* < 0.001**
Being in a relationship	26.8 (144/537)	36.8 (14/38)	27.5 (158/575)	*p* = 1.181
Having children	0.9 (5/538)	0.0 (0/40)	0.9 (5/578)	*p* = 0.540
Being a physician’s child	28.5 (157/551)	20.0 (8/40)	27.9 (165/591)	*p* = 0.248
Having (at least) one parent with a university degree	82.2 (452/550)	74.4 (29/39)	81.7 (481/589)	*p* = 0.222
Having a GP among friends or family	31.3 (171/547)	43.6 (17/39)	32.1 (188/586)	*p* = 0.111
Working experience in the social/medical sector	48.8 (270/553)	77.5 (31/40)	50.8 (301/593)	***p* < 0.001**
Completed vocational training in a medical profession^#^	12.6 (69/549)	35.9 (14/39)	14.1 (83/588)	***p* < 0.001**
Mainly grew up in ● Big city ● Small town ● Rural area	42.3 (232/549) 31.9 (175/549) 25.9 (142/549)	21.6 (8/37) 35.1 (13/37) 43.2 (16/37)	41.0 (240/586) 32.1 (188/586) 27.0 (158/586)	***p* = 0.022**
Can imagine working in** ● Big city ● Small town ● Rural area	74.5 (412/553) 66.9 (370/553) 28.4 (157/553)	17.5 (7/40) 72.5 (29/40) 97.5 (39/40)	70.7 (419/593) 67.3 (399/593) 33.1 (196/593)	***p* < 0.001** *p* = 0.467 ***p* < 0.001**

*unless otherwise indicated. **multiple answers possible. ^#^In Germany, many medical professions are three-year vocational training programs, e.g., nursing, speech therapy, physiotherapy. While most medical students start their medical studies directly after graduating from high school, a smaller proportion of first-year students have already started or completed training. Completed training is taken into account positively in the selection process.

However, rural doctor quota students were on average one year older than their fellow students, grew up almost twice as often in rural areas and less frequently in big cities. They much more often had pre-existing work experience in the medical or social sector and they had considerably more often completed vocational training in a medical profession.

Rural doctor quota students were significantly more likely to imagine working in rural areas later on but did not differ significantly in imagining working in small towns.

**Figure 1.**
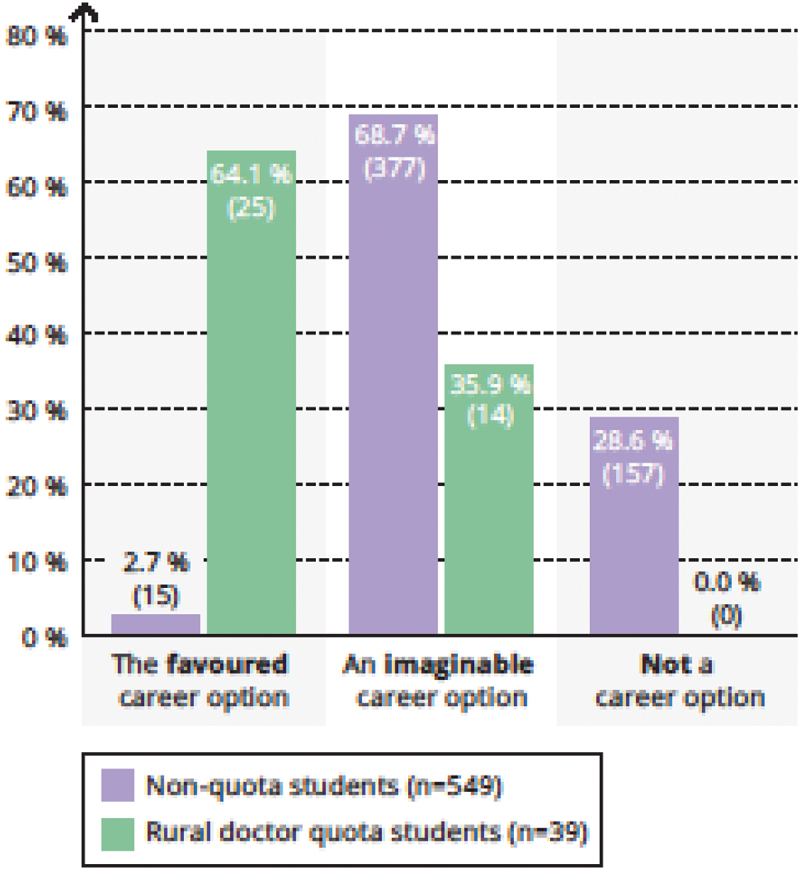
For me, general practice is …

Legend: Chi-square-test *p* < 0,001.

General practice was the *preferred* career option for 64.1% (25/39) of rural doctor quota students (vs. 2.7% [15/549] of non-quota students), an *imaginable* career option for 35,9% (14/39) and no rural doctor quota students opted for ‘not a career option’ (Figure 1).

Of those 14 rural doctor quota students who stated that general practice was an *imaginable* career option, four students named general practice or internal medicine (which also entitles the holder to set up as a GP in Germany) as their first career option in a free text ranking list. However, nine quota students named other specialties, such as pediatrics, dermatology, surgery and psychiatry as their first choice career option, of whom seven included general practice on a lower ranking in their preference list. One undecided student did not answer the free-text question.

**Figure 2.**
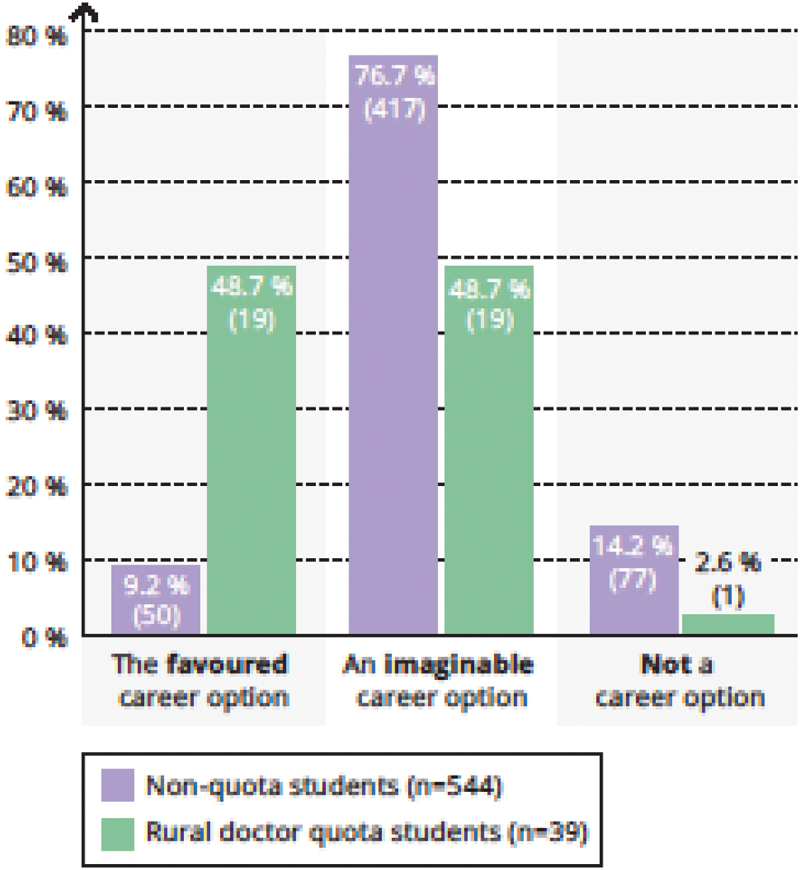
For me, working in the outpatient care sector is …

Legend: Chi-square-test *p* < 0,001.

**Figure 3.**
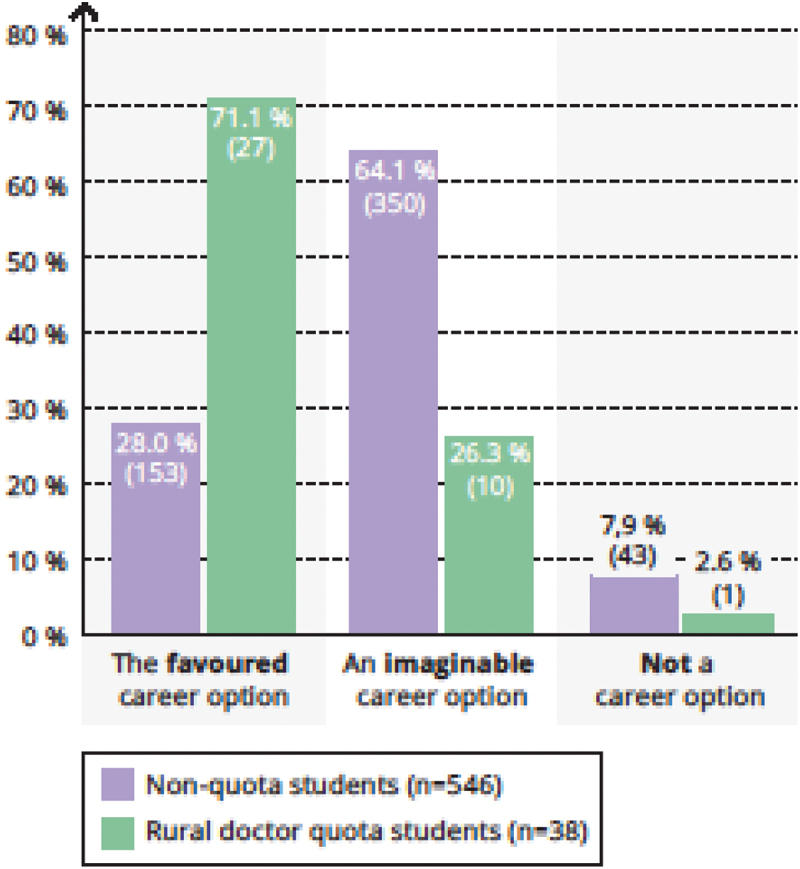
For me, working in one’s own medical practice is ….

Legend: Chi-square-test *p* < 0,001.

Working in the outpatient care sector was a preferred career option for 48.7% (19/39 of rural doctor quota students (vs. 9.2% [50/544] of non-quota students, Figure 2) and working self-employed in one’s own medical practice was the preferred option for 71.1% (27/38) of rural doctor quota students (vs. 28.0% [153/546] of non-quota students, Figure 3).

**Figure 4.**
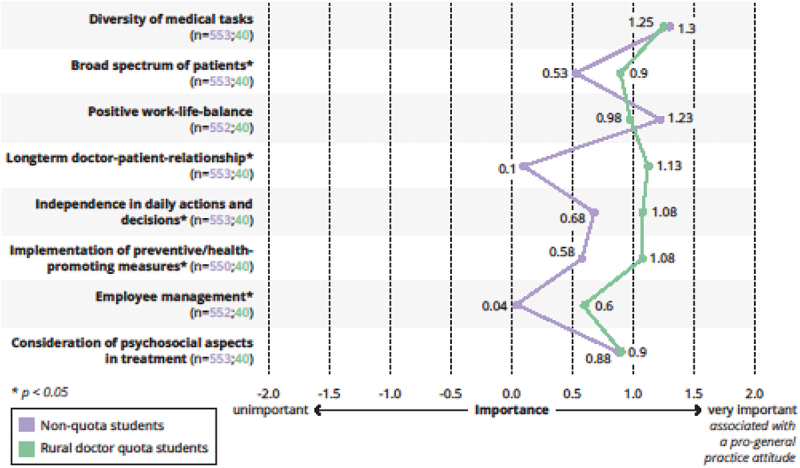
Importance of different aspects in relation to future work as a medical specialist (pro general practice).

Legend: n(non-rural doctor students/students in the rural doctor quota) are indicated for the respective item. Mann-Whitney-U-Test, *p < 0,05.

**Figure 5.**
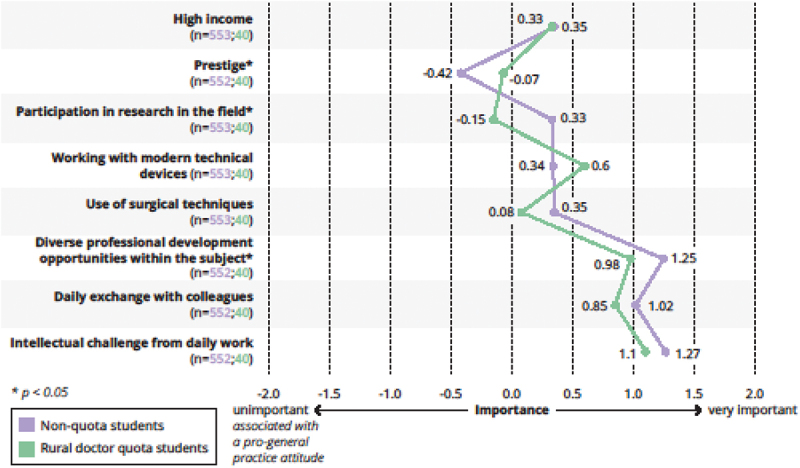
Importance of different aspects in relation to future work as a medical specialist (contra general practice).

Legend: n(non-rural doctor students/students in the rural doctor quota) are indicated for the respective item. Mann-Whitney-U-Test, *p < 0,05.

Differences in the weighting of various aspects in relation to later work, which correlate either positively or negatively with a future career decision in general practice according to current scientific literature are displayed in Figures 4 and 5.

## Discussion

### Summary of the main results

Our data analyses show that students in the rural doctor quota grew up in rural areas more often than their counterparts, had more working experience in the medical field and more often had completed vocational training. Accordingly, they were slightly older than non-quota students. Most important, the majority of these students stated that they could imagine working in the countryside. They stated that they wanted to work in general practice and aspired to be self-employed in a private practice. When asked about their career priorities, they mostly gave higher weighting to aspects that are known to be positively associated with a future career in general practice (e.g., long-term doctor–patient relationship, prevention), while they tended to give lower weighting to negatively associated aspects (involvement in research in the subject). Exceptions are discussed below.

### Literature comparison

The well-known association between rural background and the desire or readiness to work rurally [1,10] is clearly displayed in our study.

Some of our results are probably interrelated and explicable by the Saxon rural quota selection criteria: The selection process favors students with working experience in the medical sector, who are older than their counterparts who enter medical school directly after graduating from high school. Furthermore, applicants unwilling to work in rural areas are unlikely to apply for a program that requires rural residency after graduation and subsequently 10 years of work as a specialist in remote areas in Saxony. Nevertheless, our results are encouraging in that most rural quota students agree with the program objectives, even in an anonymous survey.

Notably, almost one-quarter of the rural doctor quota students did not indicate General Practice as their first choice in a free-text question, all of whom had nevertheless stated that General Practice was an imaginable career option. The rural doctor quota contract allows, in exceptional cases, to choose specialties other than general practice after applying for a permit. However, students are principally expected to qualify as GPs [20]. It is hard to imagine that the students who have gone through the application process are not aware of those requirements. Therefore, it remains unclear whether the students are flirting with the exceptional permit, are expecting the regulations to change during their studies, are prepared to subordinate their current wishes to the goals of the project in the long term, or are pursuing their own hidden goals. In conclusion, possible concerns that the new access route to medical studies might also be used by students with other career intentions cannot be ruled out completely.

Interestingly, the students in the rural doctor quota did not agree with the expectations towards future GPs on two points derived from the literature [30–33]: When asked about important aspects for their future work, ‘prestige’ was more important to them than to their counterparts, while ‘work-life balance’ is less important. This could be linked to a changing public image of general practitioners towards a more important role in the healthcare system in general, or more specifically in this group of study entrants with a rural background and corresponding experiences and role models. Based on older data, rural GPs in Germany tend to earn more than their colleagues in the city [34], but also have slightly higher working hours [34,35]. Additionally, GPs in eastern Germany (where our university is located) tend to see more patients per week than their counterparts in western Germany [35]. Accordingly, the quota students’ smaller weight on work-life-balance might display their realistic picture of the current working conditions of GPs in our area, as opposed to their non-rural oriented counterparts.

Due to the selection criteria with positive consideration of medical vocational training and work experience, it would not have been surprising if the quota students had been significantly older than their counterparts at the time of undergraduate admission. Furthermore, we know from older studies, that married and older students in particular choose to work in general practice [30–33].

Interestingly, the age of the student groups differed little and no significant differences were found in relation to being in a permanent relationship or having children (indicating an advanced stage of life).

In summary, the students selected for the rural doctor quota display many characteristics and attitudes of future rural doctors. However, some remain undecided with regard to choosing general practice as their future career, so it should not be forgotten that recruiting suitable students is only the beginning. Adapted teaching content and rural clinical experiences throughout the course are further key elements for not losing interested students over the years [36] especially as ‘rural pipeline factors’ such as rural background and rural clinical experiences might sum up [1]. Accordingly, German rural doctor quota students wished for adapted course content and experiences preparing them for their future career as rural GPs in a recent qualitative survey [28].

A longitudinal general practice curriculum already exists in Leipzig [29]. Students on the rural doctor quota were initially integrated into the existing curriculum, which will subsequently be supplemented with suitable teaching content and geared towards a stronger focus on rural medicine.

As our data shows, the quota students are a relatively inhomogeneous group. Although the students often have previous medical experience to fall back on, 2/3 have no professional experience. Accordingly, accompanying programs cannot be a ‘one fits all’ solution, but must be individually tailored. In addition, a particular need is emerging in the area of setting up a practice and long-term patient care. The students’ interest in modern technologies could be another starting point for adapted content meeting the students’ needs and preferences.

Clearly, a high quality, student-tailored, rurally focused curriculum that allows longitudinal immersion is desirable to ensure study success and for deepening and maintaining the academic goal of becoming a rural doctor.

## Strenghts and limitations

To our knowledge, this is the first survey of rural doctor quota students in Germany on their characteristics, career aspirations, and professional preferences in direct comparison to their non-quota counterparts at study entry. In contrast to the application process, our investigation was anonymous and thus potentially suitable to unmask ‘hidden’ career goals. The high response rate underpins the representativeness of the findings. As the introductory session was a formal but voluntary event, some students might not have taken part. Some students included in the calculation of the participation rate may thus not have had the opportunity to participate. However, this would underestimate rather than overestimate the response rate.

Our central group variable (rural doctor quota student vs. non-quota student) was self-reported due to the anonymity of the survey; however, the number of 40 students who self-classified as quota students is absolutely plausible and corresponds to the overall response rate.

Furthermore, it seems unlikely that students misclassify themselves as quota students in our anonymous survey.

On the other hand, total numbers are small due to the limited number of rural doctor quota students so far. We surveyed students from one medical school in one federal state of Germany. As selection processes for rural doctor quotas vary between federal states, this further limits generalizability.

Since we do not know the field of applicants, we do not actually know to what extent the differences between quota students and non-quota students are due to the admission procedure or the applicants’ self-selection. It seems plausible to us that the program mainly appeals to applicants who are biographically familiar with a life in rural areas, although, unfortunately, we do not have the relevant data.

Career choice is known to be a dynamic process in the course of medical studies. Further research is needed to monitor career goals over time. It should be kept in mind that our results may be influenced by social desirability despite the survey being anonymous. A continuation of the survey over several cohorts is desirable in order to achieve greater significance of the results through higher case numbers.

## Conclusions

In summary, our data indicate that the rural doctor quota admits students with characteristics that are associated in the literature with future rural medical practice. Despite them differing from their counterparts in orientation towards their career goal of rural GPs, rural background and working experience in the medical field, some of which are related to program prerequisites, there is little difference in other sociodemographic items such as age and marital status. Since not everyone has a fixed career goal as a rural GP despite their commitment, it is necessary to further encourage students for their future career during their studies. Our data can be a starting point here for specific support and assistance services for students who have selectively gained access to undergraduate medical training.

In Germany, the rural doctor quota has only been established for some years and further studies should address the following questions:

- How do career aspirations of rural doctor quota students develop over time, taking into account their attitudes towards General Practice at study entry?
- Can the characteristics of the rural doctor quota students be explained by the selection process alone, or do the program characteristics already appeal specifically to students with a suitable profile?
- How good is the selection procedure in terms of predicting academic success and how high are drop-out rates compared to non-quota students?
- How many rural doctor quota students leave the program during or after their studies despite becoming medical professionals due to the strict requirements?

This underpins the need for longitudinal research with data on the study progress of the program participants and on their future career choice. Since selective admission criteria to increase the number of physicians practicing in underserved areas are to be established in many countries, our results are of international interest in particular for political stakeholders, curriculum development, and undergraduate medical teachers.

## Data Availability

The datasets generated and/or analysed during the current study are not publicly available but are available from the corresponding author on reasonable request.
